# Herb-Drug Interaction: Effects of Relinqing® Granule on the Pharmacokinetics of Ciprofloxacin, Sulfamethoxazole, and Trimethoprim in Rats

**DOI:** 10.1155/2016/6194206

**Published:** 2016-09-04

**Authors:** Yuan Lu, ZiPeng Gong, YuMin Xie, Jie Pan, Jia Sun, YueTing Li, SiYing Chen, YongJun Li, YongLin Wang, Yong Huang

**Affiliations:** ^1^Provincial Key Laboratory of Pharmaceutics in Guizhou Province, No. 9, Beijing Road, Yunyan District, Guiyang 550004, China; ^2^School of Pharmacy, Guizhou Medical University, No. 9, Beijing Road, Yunyan District, Guiyang 550004, China; ^3^National Engineering Research Center of Miao's Medicines, No. 9, Beijing Road, Yunyan District, Guiyang 550004, China; ^4^Engineering Research Center for the Development and Application of Ethnic Medicine and TCM, Ministry of Education, No. 9, Beijing Road, Yunyan District, Guiyang 550004, China

## Abstract

Relinqing granule (RLQ) is the best-selling Chinese patent drug for treatment of urinary system diseases. In this study, the effects of RLQ on the pharmacokinetics of ciprofloxacin, sulfamethoxazole, and trimethoprim in SD rats were investigated. Rats were randomly divided into control group 1, control group 2, RLQ group 1, and RLQ group 2. RLQ group 1 and RLQ group 2 were treated orally with RLQ for 7 days, and rats were treated with the same volume of water in control group 1 and control group 2. Then, RLQ group 1 and control group 1 were given intragastrically ciprofloxacin on day 8, while RLQ group 2 and control group 2 were given intragastrically sulfamethoxazole and trimethoprim on day 8. Blood samples were collected and determined. There was no significant influence of pharmacokinetic parameters of trimethoprim on two groups. But some pharmacokinetic parameters of ciprofloxacin and sulfamethoxazole in RLQ pretreated rats were evidently altered (*P* < 0.05), which indicated that absorption of ciprofloxacin and sulfamethoxazole in RLQ pretreated rats was significantly affected. It indicated the coadministration of RLQ would have an influence on the efficacy of ciprofloxacin and sulfamethoxazole, and the doses of ciprofloxacin tablet and compound sulfamethoxazole tablet need adjustment.

## 1. Introduction

Relinqing granule (RLQ) is the best-selling Chinese patent drug for treatment of urinary system diseases. This product is brown to dark brown granules, with aroma smell and sweet taste, and slightly astringent. RLQ is made of aqueous extracts of the whole* Polygonum capitatum* plant and some other excipients, which has been approved by the Chinese State Food and Drug Administration [1].

*Polygonum capitatum *(*P. capitatum*) [Chinese name: Tou-hua-liao, Latin name:* Polygonum capitatum* Buch.-Ham. ex D. Don], a well-known Miao's medicinal plant, belongs to the Polygonaceae family and has been used for many years in traditional medicine, where it plays an important role in the treatment of various urological disorders, including urinary calculus and urinary tract infections [2]. Aqueous extracts of* P. capitatum *mainly included fatty acid esters, triterpenoids, steroids, flavonoids, gallic acid, and its analogs, as well as other phenolic compounds [3–9], which possessed antibacterial, anti-inflammatory, hypothermic, analgesic, antioxidant, and diuretic activities [10, 11]. Although drug therapy of RLQ alone is a feasible and effective treatment in urinary system infection patients [12, 13], it is commonly coadministered with antibiotics to enhance treatment of urinary tract infections [14–18]. Moreover, it also shows a good therapeutic effect for female acute urocystitis and chronic bacterial prostatitis in recent years [19, 20].

Ciprofloxacin is useful for the treatment of a number of bacterial infections, which belongs to a second-generation fluoroquinolone antibiotic [21]. Its spectrum of activity includes most strains of bacterial pathogens responsible for urinary tract infections. Ciprofloxacin is used alone or in combination with other antibacterial drugs in the empiric treatment of infections for which the bacterial pathogen has not been identified. Sulfamethoxazole is a sulfonamide bacteriostatic antibiotic. It is also commonly used to treat urinary tract infections. However, sulfamethoxazole is not used alone and it is always combined with trimethoprim as common usage, in therapeutic regimes for the treatment of uncomplicated acute urinary tract infections. Moreover, it is usually used as part of a synergistic combination with trimethoprim in a 5 : 1 ratio in cotrimoxazole. Consequently, RLQ combined with ciprofloxacin hydrochloride tablets or RLQ combined with compound sulfamethoxazole tablets to treat urinary tract infections are prevalent in China.

Our previous study indicated that CYP450 activity was induced by treatment with* P. capitatum* water extract [22]. However, it is well known that CYP450 enzymes are essential for the metabolism of many medications, including the majority of antibiotics [23]. Theoretically, the combination of RLQ with ciprofloxacin hydrochloride tablets or compound sulfamethoxazole tablets may lead to herb-drug interaction, while the occurrence of herb-drug interactions may affect the antibiotic activities of ciprofloxacin and sulfamethoxazole. Therefore, this study was designed to investigate the effect of RLQ on the pharmacokinetic parameters of ciprofloxacin, sulfamethoxazole, and trimethoprim.

## 2. Materials and Methods

### 2.1. Materials

Ciprofloxacin (product number 20130326), sulfamethoxazole (product number 20130406), and trimethoprim (product number 20130609) were purchased from Dalian Meilun Biology Technology Co., Ltd. (Liaoning, China); the internal standard (IS), phenacetin (product number 81105), was obtained from Dr. Ehrenstorfer GmbH (Germany). Ciprofloxacin hydrochloride tablets (250 mg per tablet) were produced by Guangzhou Bai Yun Shan Pharmaceutical General Factory (Guangzhou, China). Compound sulfamethoxazole tablets (sulfamethoxazole 400 mg and trimethoprim 80 mg per tablet) were produced by PKU International Healthcare Group Southwest Pharmaceutical Co., Ltd. (Chongqing, China). Relinqing granules were produced by Guizhou Warmen Pharmaceutical Co., Ltd. (Guizhou, China). High-performance liquid chromatography- (HPLC-) grade acetonitrile and formic acid were supplied by Merck (Darmstadt, Germany). Distilled water was obtained from Watsons Group Co., Ltd. (Hong Kong). All other reagents were of analytical grade and obtained from Kermel Technology Co., Ltd. (Tianjin, China).

### 2.2. Animals

24 male Sprague-Dawley rats (weighing 200–240 g) were provided by the Animal Supply Center of Guiyang Medical University (Certificate number SCXK2013-001, Guiyang, China). All animals maintenance and experimental study were based on the guidelines of the National Institutes of Health for the Care and Use of Animals, as well as under the approval of the Experiment Animal Center of Guiyang Medical University. All rats were kept in house under controlled temperature (22–24°C) and relative humidity (55–60%). Animals are maintained on a 12 h light/dark cycle and free access to food and water. They were fasted for 12 h, but they can drink freely before experiment.

### 2.3. Pharmacokinetic Interactions

#### 2.3.1. RLQ and Ciprofloxacin Hydrochloride Tablets

Twelve male rats were randomly divided into 2 groups (*n* = 6 for each group). RLQ group 1: there was oral administration of RLQ (4.0 g/kg twice a day; dissolved in water). Control group 1: rats were treated with the same volume of water twice daily for 7 continuous days. Fasting was carried out with free access to water on day 7, and oral administration of 0.108 g/kg ciprofloxacin (suspended in 0.5% CMC-Na) was conducted on day 8. Then, 0.15 mL of blood obtained from the caudal vein was collected at 5 min, 15 min, 30 min, 1 h, 1.5 h, 2 h, 2.5 h, 3 h, 4 h, 6 h, 8 h, and 12 h. The samples were injected into centrifuge tubes with heparin and centrifuged at 5000 rpm for 5 min. 50 *μ*L of rat plasma samples was collected and frozen at −80°C prior to analysis.

#### 2.3.2. RLQ and Compound Sulfamethoxazole Tablets

Twelve male rats were randomly divided into 2 groups (*n* = 6 for each group). RLQ group 2: there was oral administration of the RLQ (4.0 g/kg twice a day; dissolved in water). Control group 2: rats were treated with the same volume of water twice daily for 7 continuous days. Fasting was performed on the evening of day 7 with free access to water. Oral administration of 0.13 g/kg sulfamethoxazole and 0.026 g/kg trimethoprim (suspended in 0.5% CMC-Na) was carried out on day 8. Then, 0.15 mL of blood was collected at 5 min, 15 min, 30 min, 45 min, 1 h, 1.5 h, 2 h, 3 h, 4 h, 6 h, 12 h, 24 h, and 36 h and centrifuged to obtain plasma. All samples were frozen at −80°C before analysis.

### 2.4. Ultrahigh-Performance Liquid Chromatography-Mass Spectrometry (UPLC-MS)

The chromatographic analysis was performed using an ACQUITY UPLC*™* system (Waters Corp., Milford, MA, USA). A Waters TQD Quantum triple-quadrupole mass spectrometer equipped with an electrospray ionization (ESI) source was used for mass analysis and detection. Chromatographic separation was achieved on a Waters Acquity BEH C18 column (2.1 × 50 mm i.d., 1.7 *μ*m, Waters, Wexford, Ireland).

The retention times of trimethoprim, sulfamethoxazole, and phenacetin (IS) were 1.10, 1.42, and 1.54 min, respectively. The mean recoveries of ciprofloxacin, sulfamethoxazole, and trimethoprim were between 86 and 110%, and the intraday and interday precision were less than 10%. Besides, ciprofloxacin, sulfamethoxazole, and trimethoprim in analyzed samples were stable within 6 h at room temperature, 30 days at −20°C, and three freeze-thaw cycles. All validation experiments of these methods met the requirements of the Guidance for Industry Bioanalytical Method Validation Document of the American Food and Drug Administration (FDA). For ciprofloxacin, the calibration curve was linear over the concentration range from 0.125 *μ*g/mL to 25 *μ*g/mL, and the regression equation was *Y* = 6.5217*X* − 0.0503 with the mean correlation coefficient of 0.9939. The concentration ranges for sulfamethoxazole and trimethoprim were 0.1~500 *μ*g/mL and 0.1~30 *μ*g/mL, respectively, and the regression equations *Y* = 0.027*X* + 0.086 and *Y* = 1.9878*X* + 0.0716 were with the mean correlation coefficient of 0.9994 and 0.9991.

### 2.5. Ciprofloxacin Analysis

For ciprofloxacin analysis, 50 *μ*L plasma was transferred to 1.5 mL centrifuge tube, and 10 *μ*L of internal standard working solution (phenacetin, 0.76 *μ*g/mL in methanol) and 50 *μ*L of 3 mol/L formic acid were added. The mixture was vortexed for 1.0 min and then 300 *μ*L of methanol was added. This was then vortexed for another 1.0 min and centrifuged at 20,000 ×g for 10 min. The upper organic phase was transferred into tubes and evaporated to dryness under a gentle stream of nitrogen at 48°C. The residue was dissolved in 400 *μ*L of the mobile phase and centrifuged at 15000 rpm for 5 min, and 1 *μ*L of the solution was injected into UPLC-MS.

Analysis was carried out with an elution gradient of (A) acetonitrile and (B) water (both containing 0.1% formic acid) at a flow rate of 0.35 mL/min as follows: 0–2.5 min (5–95% A) and 2.5–3.5 min (95–5% A). The column and autosampler were maintained at 45°C. The injection volume was 1 *μ*L.

ESI(+) was selected as an ionization source, and the detection was conducted using selected ion recording mode (SIR), *m*/*z* 332.4 for ciprofloxacin and *m*/*z* 180.2 for phenacetin. Cone voltage was 35 V for ciprofloxacin and 30 V for phenacetin. The mass spectrometer was operated in positive mode. Desolvation temperature was set to 350°C; nebulizer gas (N_2_) and source heater were adjust to 650 L/h and 120°C, respectively. The scan time for each analyte was set at 0.05 s. Data acquisition and processing were conducted on Masslynx*™* v4.1 and Quanlynx*™* v4.1 software (Waters Corp., Milford, MA, USA).

### 2.6. Sulfamethoxazole and Trimethoprim Analysis

Plasma sample pretreatment method and gradient elution condition were the same as those used for ciprofloxacin analysis. And the settings of MS detector were the same as those used for ciprofloxacin analysis. The selected ion to sulfamethoxazole was *m*/*z* 254.3, trimethoprim was *m*/*z* 291.3, and phenacetin was 180.2. The cone voltage was 25 V for sulfamethoxazole, 35 V for trimethoprim, and 30 V for phenacetin.

### 2.7. Statistical Analysis

Data were presented as mean values ± standard deviation (SD). Pharmacokinetic parameter calculations were carried out using the DAS 2.0 pharmacokinetic program (Mathematical Pharmacology Professional Committee of China, Shanghai, China) and generated by a noncompartmental model (statistical moment). Statistically significant differences in the pharmacokinetic parameters of RLQ groups and control groups were assessed by one-way analysis of variance (ANOVA) followed by Tukey test with the level of statistical significance setting at 0.05.

## 3. Results

**Figure 1:**
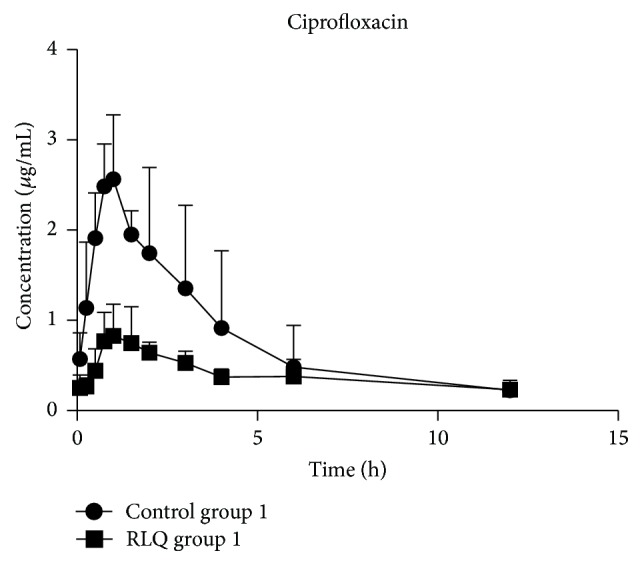
The mean concentration-time curve of ciprofloxacin in RLQ group 1 and control group 1 (*n* = 6).

The mean concentration-time curve of ciprofloxacin combined alone (control) and after repeated administration of RLQ is shown in Figure 1, and corresponding pharmacokinetic parameters are shown in Table 1. Compared with the control group 1, the RLQ group 1 had *t*_1/2*z*_, CL_*z*_/*F*, and *V*_*z*_/*F* that were ×1.86, ×1.89, and ×3.17 higher; AUC_(0–*∞*)_ and *C*_max_ decreased by 50.0% and 29.3% (*P* < 0.05), respectively. These results indicated that preadministration of 4.0 g/kg RLQ (twice a day) substantially changed the pharmacokinetics of ciprofloxacin in rats, leading to the reduction of the ciprofloxacin AUC_(0–*t*)_, AUC_(0–*∞*)_, and *C*_max_ and the simultaneous elevation of *t*_1/2*z*_, CL_*z*_/*F*, and *V*_*z*_/*F*.

**Figure 2:**
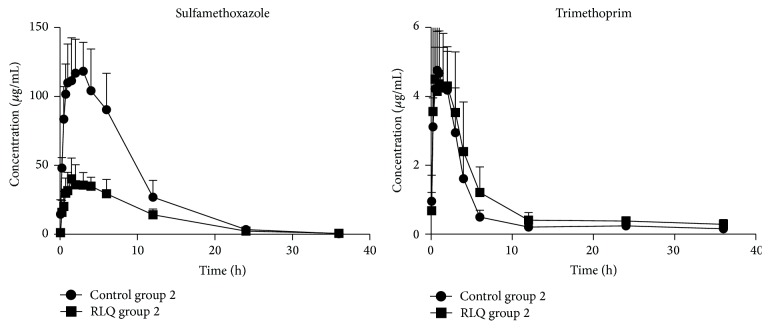
Mean plasma concentration-time curves of sulfamethoxazole and trimethoprim in RLQ group 2 and control group 2 (*n* = 6).

The mean concentration-time curves of sulfamethoxazole and trimethoprim alone (control) and after repeated administration of RLQ are shown in Figure 2, and corresponding pharmacokinetic parameters are shown in Table 2. Compared with the control group 2, RLQ group 2 showed CL_*z*_/*F* and *V*_*z*_/*F* for sulfamethoxazole were ×2.50 and ×2.94 higher and AUC_(0–*∞*)_ and *C*_max_ decreased by 36.79% and 35.79% (*P* < 0.05), respectively. However, there were no substantial differences in relevant parameters of trimethoprim between control group 2 and RLQ group 2. Results from the pharmacokinetic experiment showed that preadministration of RLQ significantly influenced the pharmacokinetics of sulfamethoxazole in rats, leading to the elevation of the sulfamethoxazole CL_*z*_/*F* and *V*_*z*_/*F* and the reduction of AUC_(0–*∞*)_ and *C*_max_.

## 4. Discussion and Conclusions

The pharmacokinetics of drugs includes absorption, distribution, metabolism, and excretion, and any effects on these processes will alter the way in which the drug acts upon the body. In this study, AUC and *C*_max_ of ciprofloxacin in RLQ group 1 significantly decreased; simultaneously, *t*_1/2*z*_, CL_*z*_/*F*, and *V*_*z*_/*F* in RLQ group 1 were significantly increased. AUC_(0–*∞*)_ and *C*_max_ of sulfamethoxazole were decreased significantly, as compared with control group 2. In addition, CL_*z*_/*F* and *V*_*z*_/*F* were significantly increased in RLQ group 2. Pretreatment with RLQ decreased the bioavailability of ciprofloxacin and sulfamethoxazole after oral administrations for 7 continuous days but did not lead to accelerating of the metabolism of those antibiotics. Hence, it is proposed that the activity of P-gp further reduced the absorption of ciprofloxacin/sulfamethoxazole, which led to drug interactions. This was confirmed in Figures 1 and 2, which showed that the ciprofloxacin and sulfamethoxazole plasma concentration of the RLQ preadministration groups was significantly reduced compared with that of the control group. The effect of RLQ on trimethoprim looks negligible, which has been confirmed by the whole pharmacokinetic parameters observed in the present study.

The results of the previous research showed that* Polygonum capitatum* could induce CYP2C9 and CYP3A4 and did not influence CYP1A2, CYP2C19, or CYP2E1 [23]. According to previous reports, sulfamethoxazole is eliminated mainly by metabolism, and CYP2C9 plays an important role in its N_4_-hydroxylation [24]. However, the rats in the RLQ group 2 had no substantial changes in *t*_1/2_ when compared with the control group. Intestinal secretory movement of ciprofloxacin may limit its oral bioavailability, because it is a P-gp substrate [25, 26]. So we can extrapolate that the changes of ciprofloxacin and sulfamethoxazole pharmacokinetics were negligible to P450-induced oxidation reaction but prominent to P-gp induced reaction.

In summary, this study found that RLQ decreased the bioavailability of ciprofloxacin and sulfamethoxazole but did not affect the bioavailability of trimethoprim in SD rats.

It is well known that antibiotics can exert their therapeutic effect when their plasma-drug concentration is maintained above the minimal inhibitory concentration (MIC). If the plasma concentration of antibiotic goes down lower than the MIC, it will possibly lead to failure of therapy even drug resistance. RLQ reduced plasma concentrations of sulfamethoxazole and ciprofloxacin, which were combined clinically with RLQ in common usage. It would perhaps lead to reducing antibacterial action of ciprofloxacin and cotrimoxazole (coadministration of trimethoprim and sulfamethoxazole). Therefore, clinicians should adjust the dosage of ciprofloxacin and cotrimoxazole in patients who take RLQ.

## Figures and Tables

**Table 1:** Main pharmacokinetic parameters of ciprofloxacin alone (control) and after repeated administration of RLQ (*n* = 6).

Parameters	Control group 1	RLQ group 1
AUC_(0–*∞*)_ (mg/L*∗*h)	11.75 ± 4.35	5.88 ± 1.28^*∗*^
*t*_1/2__*z*_ (h)	3.16 ± 1.29	5.89 ± 2.24^*∗*^
*T*_max_ (h)	1.58 ± 0.49	1.67 ± 0.52
CL_*z*_/*F* (L/h/kg)	10.14 ± 3.14	19.12 ± 4.18^*∗∗*^
*V*_*z*_/*F* (L/kg)	49.81 ± 31.06	155.24 ± 47.10^*∗∗*^
*C*_max_ (mg/L)	3.52 ± 1.32	1.03 ± 0.30^*∗∗*^

^*∗*^ *P* < 0.05 when compared with related parameters of control group 1. ^*∗∗*^ *P* < 0.01 when compared with related parameters of control group 1.

**Table 2:** Main pharmacokinetic parameters of sulfamethoxazole and trimethoprim alone (control) and after repeated administration of RLQ.

Parameters	Sulfamethoxazole		Trimethoprim	
	Control group 2	RLQ group 2	Control group 2	RLQ group 2
AUC_(0–*∞*)_ (mg/L*∗*h)	1205.36 ± 330.02	443.46 ± 55.36^*∗∗*^	27.31 ± 12.61	37.28 ± 14.01
*t*_1/2__*z*_ (h)	4.26 ± 0.87	4.92 ± 0.76	19.01 ± 10.68	14.11 ± 5.62
*T*_max_ (h)	2.0 ± 0.75	2.58 ± 1.31	1.21 ± 0.62	1.3 ± 1.00
CL_*z*_/*F* (L/h/kg)	0.2 ± 0.07	0.5 ± 0.06^*∗∗*^	9.18 ± 3.34	6.77 ± 3.15
*V*_*z*_/*F* (L/kg)	1.21 ± 0.53	3.56 ± 0.66^*∗∗*^	279.34 ± 256.73	124.42 ± 38.34
*C*_max_ (mg/L)	129.14 ± 18.39	46.22 ± 9.58^*∗∗*^	4.96 ± 1.12	5.6 ± 2.05

^*∗∗*^ *P* < 0.01 when compared with related parameters of control group 2.

## References

[1] Liao S.-G., Zhang L.-J., Sun F. (2011). Antibacterial and anti-inflammatory effects of extracts and fractions from *Polygonum capitatum*. *Journal of Ethnopharmacology*.

[2] Editorial Committee of Chinese Materia Medica (2005). *State Administration of Traditional Chinese Medicine, Miao's Material Medica, Chinese Materia Medica (Zhonghua Bencao)*.

[3] Li Y. M., Gong Y. (2007). The research progress on the chemical component and the pharmacology of *Polygonum capitatum* Ham ex D. Don. *Journal of Guizhou University (Natural Sciences)*.

[4] Liu Z.-J., Qi J., Zhu D.-N., Yu B.-Y. (2008). Chemical constituents from Polygonum capitatum and their antioxidation activities in vitro. *Journal of Chinese Medicinal Materials*.

[5] Yu M., Li Z. L., Li N. (2008). Chemical constituents of the aerial parts of *Polygonum capitatum*. *Journal of Shenyang Pharmaceutical University*.

[6] Yang Y., Cai F., Yang Q., Yang Y.-B., Sun L.-N., Chen W.-S. (2009). Study on chemical constituents of *Polygonum capitatum* Buch.-Ham. ex D. Don (I). *Academic Journal of Second Military Medical University*.

[7] Zhang L. J., Liao S. G., Zhan Z. H. (2010). A study on the phenolic constituents of *Polygonum capitatum*. *Lishizhen Medicine and Materia Medica Research*.

[8] Zhao H. X., Bai H., Wang Y. S. (2010). Progress on chemical constituents and analytical methods of *Polygonum capitatum* Buch.-Ham. Ex D. Don. *Food and Drug*.

[9] Liao S.-G., Zhang L.-J., Wang Z. (2012). Electrospray ionization and collision-induced dissociation tandem mass spectrometric discrimination of polyphenolic glycosides: exact acylation site determination of the O-acylated monosaccharide residues. *Rapid Communications in Mass Spectrometry*.

[10] Ren G., Chang F., Lu S., Zhong H., Zhang G. (1995). Pharmacological studies of *Polygonum capitatum* Buch-Ham. ex D. Don. *China Journal of Chinese Materia Medica*.

[11] Liu M., Luo C. L., Zhang Y. P. (2007). Analgesic, anti-inflammatory, and diuretic effects of *Polygonum capitatum* and *Toddalia asiatica*. *Guizhou Medical Journal*.

[12] Yang L. M., Bai M. J., Zhang Y. (2002). A clinical observation: relinqing granules in treatment of urinary tract infection. *Acedemic Periodical of Changchun College of Traditional Chinese Medicine*.

[13] Chen J. W., Tan X., Miao Z. R. (2007). Clinical observation of Relinqing granules for the treatment of male nongonococcal urethritis in 60 cases. *China Pharmaceuticals*.

[14] Xia L., Zhang Z., Han L. (2004). Clinical observation of Relinqing granules and doxycycline combined for the treatment of nongonococcal urethritis. *Journal of Clinical Urology*.

[15] Ou Yang X. R., Min Y. G., Jiang W. X., Guo Z. X. (2006). Clinical observation of Relinqing granules and clarithromycin combined treating nongonococcal urethritis. *Journal of Dermatology and Venereology*.

[16] Li G. Q., Wang L. P., Chen M. C. (2007). Clinical observation of Relinqing granules and gatifloxacin combined for the treatment of nongonococcal urethritis in 62 cases. *Southern China Journal of Dermato-Venereology*.

[17] Hong K., Yuan R. P., Jiang H. (2009). A randomized study comparing combined Relinqing and azithromycin for the treatment of nongonococcal urethritis in men. *The Chinese Journal of Human Sexuality*.

[18] Su Z. R., Liu L. Q. (2013). Clinical observation of Relinqing granules and levofloxacin combined treating urinary system infection. *Strait Pharmaceutical Journal*.

[19] Zhang W. H., Hu L. F., Zhang R. (2011). Clinical observation of Relinqing granules and galtixacin combined for the treatment of chornic bacterial prostatitis. *Medical Journal of National Defending Forces in Northwest China*.

[20] Ma J., Wu J. F., Lan X. X. (2012). Clinical observation of Relinqing granules and lomefloxacin combined for the treatment of female acute urocystitis in 36 cases. *National Medical Frontiers of China*.

[21] Oliphant C. M., Green G. M. (2002). Quinolones: a comprehensive review. *American Family Physician*.

[22] Zheng L., Lu Y., Cao X. (2014). Evaluation of the impact of *Polygonum capitatum*, a traditional Chinese herbal medicine, on rat hepatic cytochrome P450 enzymes by using a cocktail of probe drugs. *Journal of Ethnopharmacology*.

[23] Theuretzbacher U., Zeitlinger M. (2011). Antibacterial distribution and drug-drug interactions in cancer patients. *Principles and Practice of Cancer Infectious Diseases*.

[24] Cribb A. E., Spielberg S. P., Griffin G. P. (1995). N4-hydroxylation of sulfamethoxazole by cytochrome P450 of the cytochrome P4502C subfamily and reduction of sulfamethoxazole hydroxylamine in human and rat hepatic microsomes. *Drug Metabolism and Disposition*.

[25] Haslam I. S., Wright J. A., O'Reilly D. A., Sherlock D. J., Coleman T., Simmons N. L. (2011). Intestinal ciprofloxacin efflux: the role of breast cancer resistance protein (ABCG2). *Drug Metabolism and Disposition*.

[26] Leitner I., Nemeth J., Feurstein T. (2011). The third-generation P-glycoprotein inhibitor tariquidar may overcome bacterial multidrug resistance by increasing intracellular drug concentration. *Journal of Antimicrobial Chemotherapy*.

